# Susceptibility to *Plasmodium vivax* malaria associated with DARC (Duffy antigen) polymorphisms is influenced by the time of exposure to malaria

**DOI:** 10.1038/s41598-018-32254-z

**Published:** 2018-09-14

**Authors:** Flora Satiko Kano, Aracele Maria de Souza, Leticia de Menezes Torres, Marcelo Azevedo Costa, Flávia Alessandra Souza-Silva, Bruno Antônio Marinho Sanchez, Cor Jesus Fernandes Fontes, Irene Silva Soares, Cristiana Ferreira Alves de Brito, Luzia Helena Carvalho, Tais Nobrega Sousa

**Affiliations:** 1 Molecular Biology and Malaria Immunology Research Group, Instituto René Rachou, Fundação Oswaldo Cruz (FIOCRUZ), Belo Horizonte, Minas Gerais Brazil; 2 Departamento de Engenharia de Produção, Universidade Federal de Minas Gerais, Belo Horizonte, Minas Gerais Brazil; 3 Universidade Federal de Mato Grosso, Cuiabá, Mato Grosso Brazil; 4 Hospital Julio Muller, Universidade Federal de Mato Grosso, Cuiabá, Mato Grosso Brazil; 5 Departamento de Análises Clínicas e Toxicológicas, Faculdade de Ciências Farmacêuticas, Universidade de São Paulo, São Paulo, SP Brazil

## Abstract

Malaria has provided a major selective pressure and has modulated the genetic diversity of the human genome. The variants of the Duffy Antigen/Receptor for Chemokines (DARC) gene have probably been selected by malaria parasites, particularly the FY*O allele, which is fixed in sub-Saharan Africa and confers resistance to *Plasmodium vivax* infection. Here, we showed the influence of genomic ancestry on the distribution of DARC genotypes in a highly admixed Brazilian population and confirmed the decreased susceptibility of the FY*A/FY*O genotype to clinical *P. vivax* malaria. FY*B/FY*O individuals were associated with a greater risk of developing clinical malaria. A remarkable difference among DARC variants concerning the susceptibility to clinical malaria was more evident for individuals who were less exposed to malaria, as measured by the time of residence in the endemic area. Additionally, we found that DARC-negative and FY*A/FY*O individuals had a greater chance of acquiring high levels of antibodies against the 19-kDa C-terminal region of the *P. vivax* merozoite surface protein-1. Altogether, our results provide evidence that DARC polymorphisms modulate the susceptibility to clinical *P. vivax* malaria and influence the naturally-acquired humoral immune response to malaria blood antigens, which may interfere with the efficacy of a future vaccine against malaria.

## Introduction

*Plasmodium vivax* is the most widespread malaria parasite species outside Africa, accounting for 64% of malaria cases in endemic regions of the Americas, greater than 30% of cases in South East Asia and 40% of cases in the Eastern Mediterranean regions^1^. Moreover, malaria caused by *P. vivax* is no longer considered a benign disease because of the reports of deadly malaria caused by this parasite^2–5^.

To establish erythrocyte infection in humans, *P. vivax* is highly dependent on the interaction of the parasite Duffy binding protein (region II, PvDBP_II_) and its cognate receptor in the surface of reticulocytes, Duffy antigen receptor for chemokines (DARC) (recently renamed Atypical chemokine Receptor 1)^6–8^. The *DARC* gene has three common alleles, FY*A, FY*B and FY*O or FY*B^*ES*^ (also known as Duffy negative)^9–11^. The two functional alleles, FY*A and FY*B, are the result of one non-synonymous mutation (125 G > A, rs12075) and are associated with the Fy^a^ and Fy^b^ blood-group antigens, respectively^9^. The non-functional allele FY*O is defined by a mutation in the gene promoter (−33T > C, rs2814778) that abrogates its expression in the erythrocyte cell lineage^10,11^. An investigation of the evolutionary history of DARC has estimated the age of the FY*O allele to be 42,000 years, likely older than most other mutations associated with malaria resistance^12,13^. Likewise, this ancient allele seems to have undergone fixation in Africa in a scenario in which all three common DARC alleles were segregated in this continent^12^. Therefore, the FY*O allele has been used as a marker for African ancestry. The FY*O allele has been shown to protect against *P. vivax* infection^6^. Different susceptibilities to *P. vivax* have also been associated with the Duffy blood group antigens (Fy^a^ and Fy^b^) because polymorphisms alter the binding to the parasite’s DBP, and densities of the antigen in the erythrocyte surface^14,15^.

Acquired immunity against infection and clinical protection in malaria has been somehow mediated by antibodies^16^. Antibodies against PvDBP_II_ showed a natural occurrence in individuals from *P. vivax* endemic areas^17–19^. These antibodies could block the DBP-DARC interaction, inhibit the erythrocyte invasion of *P. vivax*^20,21^ and seem to be associated with clinical protection^21,22^. Because the DARC-negative promoter heterozygously expresses approximately half of the total amount of DARC^14,23^, both the DARC antigen and its expression level may play a major role in the immunity against *P. vivax*^24,25^. We and others have shown that the frequency of naturally acquired antibodies against PvDBP_II_ is inversely correlated with the DARC expression levels^24,25^. In a population-based study in the Brazilian Amazon, we showed that the naturally acquired binding-inhibitory antibodies targeting PvDBP_II_ were more frequent in heterozygous individuals carrying the non-functional DARC allele FY*O^25^. Similarly, individuals expressing less DARC from Colombian malaria-endemic areas exhibited higher frequencies and magnitudes of anti-PvDBP_II_ IgG antibodies, as evaluated by conventional serology (ELISA-detected IgG)^24^.

In the current study, we investigated how polymorphisms in DARC modulate the susceptibility to *P. vivax* malaria in individuals differing in the levels of exposure to malaria in an agricultural settlement of the Amazon region. This population showed a high genetic admixture with a significant contribution of European and Native American ancestries, which reflected the distribution of DARC genotypes. A remarkable difference among DARC variants in the susceptibility to clinical *P. vivax* malaria was associated with low cumulative exposure to malaria, as measured by the time of residence in the endemic area. Additionally, evidence of the association between naturally acquired immune response to the 19-kDa C-terminal region of the *P. vivax* merozoite surface protein-1 (PvMSP1_19_), a leading *P. vivax* vaccine candidate, and DARC variants was provided, for the first time, in a community-based study.

## Methods

### Study Area and Population

The study was carried out in the agricultural settlement of Rio Pardo, in the Presidente Figueiredo municipality, located in the Northeast of Amazonas State. The study area and malaria transmission patterns have been described in detail elsewhere^25,26^. Briefly, the settlement was officially created in 1996 by the National Institute of Colonization and Agrarian Reform (INCRA) as part of a large-scale colonization project focused on agriculture and wide-ranging human settlement in the Amazon area^27^. The Rio Pardo population consisted of 701 inhabitants according to the census conducted between September and October 2008. Most residents were native to the Amazon region, mainly from the Amazonas State. The population lives mainly on subsistence farming and fishing along the tributaries of the Rio Pardo River.

The study area was classified as hypo- to mesoendemic based on the spleen size of the local children and parasite infection rates^26^. Malaria transmission occurs throughout the year. *P. vivax* was responsible for most cases of malaria (average of 80%) during the period of study (2003–2009) in the region. The highest number of malaria cases were in 2004 to 2006 (the average annual parasite incidence (API) was 289 cases per 1000 inhabitants), followed by a continuous decrease in the number of cases; in 2009, the API was 54.6 (Health Surveillance Secretariat of the Brazilian Ministry of Health, SVS/MS).

### Study design and cross-sectional surveys

The ethical and methodological aspects of this study were approved by the Ethics Committee of Research involving Human Subjects of Institute René Rachou/Fiocruz (Reports No. 07/2006, No. 07/2009, No. 26/2013, and No. 1.395.372), according to the Resolution of the Brazilian Council on Health-CNS 466/12. All participants signed a written informed consent, including the next of kin, caretakers, or guardians, on behalf of the minors/children enrolled in the study. All the methods were carried out in accordance with the approved guidelines.

A population-based open cohort study was initiated in November 2008, followed by two cross-sectional surveys six (June 2009) and twelve months (October-November 2009) after the initial survey. The cross-sectional surveys have been described in detail elsewhere^26^. For each resident, the number of *P. vivax* malaria episodes (mono and mixed infections with *P. falciparum*) and information about the parasite species determined by microscopy were obtained from the Epidemiological Surveillance System for Malaria (SIVEP-Malaria) for the period residing in the Rio Pardo community between 2003 (when the data were available in the database) and 2009. In Brazil, all cases of malaria are compulsorily reported to the Ministry of Health through the national database SIVEP-Malaria^28^. The study area registered a median of 321 (Range = 168–391) *P. vivax* malaria cases, 21 (Range = 5–236) *P. falciparum* malaria cases and 3 (Range = 1–13) mixed cases by these two species between 2003 and 2009.

Four hundred ninety-eight subjects were enrolled at the baseline, 495 at the 2^nd^ survey, and 495 in the 3^rd^ survey. In total, 690 subjects were successful genotyped to DARC polymorphisms. The genomic ancestry was inferred for a subset of 339 unrelated subjects (first-degree relatives). Serological assays were carried out for 207 individuals to PvDBP_II_ and 222 individuals to PvMSP1_19_ who were enrolled in the three cross-sectional surveys.

### Laboratory diagnosis of malaria

For participants at the cross-sectional surveys, malaria infection was diagnosed by microscopy of Giemsa solution-stained thick smears and real-time PCR amplification of a species specific segment of the 18S rRNA gene of human malaria parasites as previously described^29^. Briefly, real-time PCR was performed using a consensus pair of oligonucleotides (PL1473F18 [5′-TAACGAACGAGATCTTAA-3′] and PL1679R18 [5′- GTTCCTCTAAGAAGCTTT-3′]), and each 20 µL reaction mix contained 2 μL of genomic DNA (approximately 100 ng), 10 µL of Sybr Green PCR master mix (Applied Biosystems, Foster City, CA, USA), 2.5 mM MgCl_2_ and 0.5 μM of each oligonucleotide. The PCR conditions consisted of an initial denaturation step at 95 °C for 10 min, followed by 40 cycles of 20 sec at 90 °C, 30 sec at 50 °C and 30 sec at 60 °C. After amplification, the melting curves were observed from the dissociation curves resulting from continuous measurements of fluorescence at 530 nm during which the temperature was gradually increased from 60 °C to 95 °C. The amplification and fluorescence detection were performed using the ABI PRISM 7500 Sequence Detection System (Applied Biosystems).

The Giemsa solution-stained smears were evaluated by experienced microscopists, according to the malaria diagnosis guidelines of the Brazilian Ministry of Health. For real-time PCR, genomic DNA was extracted from either EDTA whole-blood samples (adults and children ≥ 5 years in age) or dried blood spots on filter paper (children < 5 years in age) using the Puregene blood core kit B (Qiagen, Minneapolis, MN, USA) or QIAmp DNA mini kit (Qiagen), respectively, according to the manufacturers’ instructions.

### Genotyping of Ancestry-Informative Markers (AIMs)

The subjects were typed for a set of 48 single-nucleotide polymorphisms (SNPs) previously validated as a useful tool for ancestry estimation in population samples from diverse origin^30^ (Supplementary Table S1). This subset of markers was chosen using the *ln* algorithm^31^ and was defined as the most ancestry-informative markers (AIMs) distinguishing one or more of the four continental populations (European, African, Native American, and East Asian). The AIMs were genotyped by real-time PCR with specific hydrolysis probes for each SNP (Applied Biosystems). The assays were performed in a total volume of 5 µL and in the presence of 2.5 µL of Taqman*®* Universal PCR Master Mix 2 × (Applied Biosystems), 0.25 µL of Genotyping Assay Mix (Applied Biosystems), 1.25 µL of water and 1 µL of DNA (approximately 10 ng). The cycling parameters for the PCR were as follows: initial denaturation at 95 °C for 10 min, 40 cycles of 15 sec at 95 °C and 1 min at 60 °C. Amplification and fluorescence detection were carried out using the ViiA 7 Real-Time PCR System (Applied Biosystems).

### Ancestry estimates

We estimated the individual ancestry using the method implemented in the program Structure 2.3.4^32,33^. The program implements a Bayesian clustering method to infer population structure and an admixture using a Markov Chain Monte Carlo (MCMC) procedure. The burn-in was set at 50,000 steps followed by 500,000 MCMC steps, and a model of correlated allele frequencies was specified. Thirty replicates were performed assuming three clusters (K = 3) because the Brazilian population was formed by an admixture from three parental populations: Native Americans, Europeans and Africans. For all runs, lambda was set to 1.0, α parameter was estimated from the data, and the priori information for the individuals from parental populations to assist the clustering was not used (USEPOPINFO = 0). All replicates were evaluated using CLUMPP^34^, and specific solutions were plotted using DISTRUCT 1.1^35^. To avoid the bias caused by the family structure in our population structure analyses, we excluded related samples that were identified using individual data of family name and household information.

The data of parental populations (European, African and Native American) were a public dataset obtained from the HGDP-CEPH Diversity Panel (http://www.hagsc.org/hgdp/files.html) and Hapmap Project (http://hapmap.ncbi.nlm.nih.gov/) and Kosoy *et al*.^30^. We downloaded all genotypes for 611 unrelated individuals, which included the following: 60 CEPH Europeans (CEU, Utah Residents with Northern and Western European ancestry), 128 European Americans (NYCPEA), 80 Yoruban Africans (Nigeria), 19 Bini West Africans (Edo State, Nigeria), 23 Kanuri West Africans (Northern Nigeria), 12 Bantus (Kenya), 31 Biaka Pygmies (Central African Republic), 15 Mbuti Pygmies (Democratic Republic of Congo), 24 Mandenka individuals (Senegal), 6 San individuals (Namibia), 75 Mayan Amerindians (Mexico and Guatemala), 25 Pimas (Mexico), 13 Colombians (Colombia), 24 Karitiana Amerindians (Brazil), 21 Surui Amerindians (Brazil), 29 Nahua Amerindians (Central Mexico), and 26 Quechuan Amerindians (Peru). The NYCPEA individuals were from the New York City, and their classification was based on the self-classification of ethnicity^36^.

Additionally, to represent the genetic structure of our samples, we performed principal component analysis (PCA) of individual genotypes, as implemented in Adegenet and Ade4 for the R environment^37^. The non-linear iterative partial least squares (NIPALS) algorithm implemented in Ade4 was applied to consider the missing values.

### DARC Genotyping

We genotyped two SNPs in the *FY* gene (−33T > C and 125 G > A) that defines the three common DARC alleles: FY*A, FY*B, and FY*O. SNPs were genotyped by real-time PCR with allele-specific oligonucleotides, as previously described^25,38^. Briefly, the 20-µL reaction mix contained 50–100 ng of genomic DNA, 10 µL of SYBR Green PCR master mix (Applied Biosystems) and 0.1–1.0 µM of each oligonucleotide (Supplementary Table S2). The amplification and fluorescence were detected using the ABI Prism 7000 Sequence Detection System (Applied Biosystems). The cycling parameters for the real-time PCR comprised a cycle of 95 °C for 10 min, followed by 35 cycles of 95 °C for 15 s and 60 °C for 1 min. After amplification, melting curves were observed from the dissociation curve. Genotyping results were confirmed for approximately 10% of samples, including all FY*O/FY*O individuals, by sequencing a 942-bp fragment of the *DARC* gene, comprising polymorphic positions −33T > C and 125 G > A, as previously described^25^.

### Serological Assays

We carried out enzyme-linked immunosorbent assay (ELISA) for total IgG antibodies against PvDBP_II_ and PvMSP1_19_. The recombinant protein PvDBP_II_ included amino acids 243–573 (region II) of the Sal-1 PvDBP_II_ variant; the recombinant protein was expressed as a 39-kDa 6 × His fusion protein, as previously described^39^. The oligonucleotides used to amplify the fragment of PvDBP_II_ by PCR were as follows: sense (5′-TTTGGATCCACGATCTCTAGTGCTATTATAAATC-3′) and anti-sense (5′-AAACTCGAGTGTCACAACTTCCTGAGTATTTTT-3′). The recombinant protein representing PvMSP1_19_, which contains amino acids 1616–1704 of the full-length MSP1 protein, was expressed as a 6× His fusion protein in *Escherichia coli*, as described elsewhere^40–42^. The PCR was performed using the sense (5′-ACCAGCCATATGACTATGAGCTCCGAGCACACA-3′) and anti-sense (5′-CGGATCCTCGCTACAGAAAACTCCCTCAAA-3′) oligonucleotides^41^. ELISA was carried out as previously described^18^ with serum samples diluted at 1:100 and PvDBP_II_ and PvMSP1_19_ at final concentrations of 5 μg/mL and 1 μg/mL, respectively. The sera from individuals naturally exposed to *P. falciparum* failed to cross-react in the ELISA assay with the same construction of the recombinant protein PvMSP1_19_ used in the present study^42^. The results were expressed as the reactivity index (RI), calculated by dividing the reading values of the test (OD values) by the cut-off (mean reading for the unexposed group plus 3 SD, n = 30). Values of RI > 1.0 were considered positive.

### Statistical Methods

A database was created using Epidata software (http://www.epidata.dk). The Kruskal–Wallis test with Dunn’s post-hoc test and the Mann-Whitney U test were performed to compare the differences in the antibody response during the follow-up period. Fisher’s exact test was performed to compare the distribution of the frequency of DARC genotypes among areas of residence in the Rio Pardo community. Associations among age, number of malaria episodes, and time of residence in the Amazon region were estimated using the Spearman’s rank correlation test and the R *cor.test* function. Because of the high correlation between age and the time of residence in the Amazon region, we used the last predictor as a measure of exposure to malaria in further association analysis. All statistical analyses were performed using R software (version 3.4). A *P*-value < 0.05 was considered significant in all analyses.

#### Genetic Population Analysis

The Hardy-Weinberg equilibrium for each SNP was calculated using the R package SNPassoc^43^. To investigate how the ancestry-based population subdivision influenced the Rio Pardo population structure, the association between homozygosity excess (as measured by *F*_IT_ for each SNP) and ancestry (estimated by *F*_ST_ for each SNP between the European and Native American population, the two main ancestry sources of the Rio Pardo population) was estimated using the Spearman’s rank correlation test^44^. The population *F*_IT_ was estimated by averaging *F*_IT_ across SNPs. We used Arlequin 3.5^45^ to calculate the observed and expected heterozygosities under Hardy–Weinberg equilibrium (*H*_O_ and *H*_E_, respectively) and then estimated *F*_IT_ = (*H*_E _− *H*_O_)/*H*_E_ for each SNP. *F*_ST_ was estimated using R package hierfstat^46^. The *F*_IS_ statistic for the Rio Pardo population was estimated using Arlequin 3.5.

#### Regression Analysis

Multinomial logistic regression was applied to describe the association between individual genomic ancestry and the frequency of DARC genotypes. The models were fitted using the *multinom* function available in the R package nnet^47^.

The logistic regression model with stepwise backward deletion was applied to describe the association between the antibody to *P. vivax* blood antigens and covariates: DARC genotype, number of slide-positive malaria episodes recorded between 2003 and 2009, recent *P. vivax* malaria infection (determined from SIVEP-Malaria and self-reported malaria episodes), time of residence in the Amazon region and place of residence in Rio Pardo community. Covariates were selected for inclusion in the logistic models if they were associated with the outcome at the 15% level of significance in exploratory unadjusted analysis. The models were fitted using the R *glm* function. Only variables associated with statistical significance at the 5% level were maintained in the final model. The goodness of fit was assessed by comparing the deviance of candidate models and by the Akaike information criteria (AIC) statistics^48^. Because of missing values, 195 of 196 observations remained in the final logistic regression model.

Zero-inflated Poisson Regression (ZIP) was carried out to assess the relationship between the number of clinical *P. vivax* malaria that each subject experienced in a period of seven years (2003–2009) and covariates: DARC genotype, time of residence in the Amazon region and place of residence in Rio Pardo community. The offset of the ZIP regression was defined as the time that an individual lived in the Rio Pardo community during the study period. ZIP regression analyses were performed using *zeroinfl* function in the R package pscl^49^. The goodness of fit was assessed by comparing the deviance of candidate models and by Akaike information criteria (AIC) statistics^48^. The candidate models were tested by leave-one-out cross-validation and we chose the model with the least cross-validation error (the generalized Pearson statistic^50^ and the sum of squared error) that indicated the best predictive model. Because of missing values, 633 of 690 observations remained in the final ZIP regression model.

#### Clustering Analysis

K-means cluster analyses were performed on the IgG total antibodies against blood-stage antigens (PvMSP1_19_ and PvDBP_II_) data using the R *cluster* function. We used the antibody response against each antigen measured in three periods (baseline, six and twelve months after the initial survey) to generate k clusters. Cluster numbers between 2 and 15 were evaluated and the k clusters with the lowest total within groups sum of squares was determined as the appropriate number of clusters. Clustering data were visualized using the *clusplot* function in the R package cluster^51^.

## Results

### Population Structure in the Amazonian Settlement of Rio Pardo

**Figure 1:**
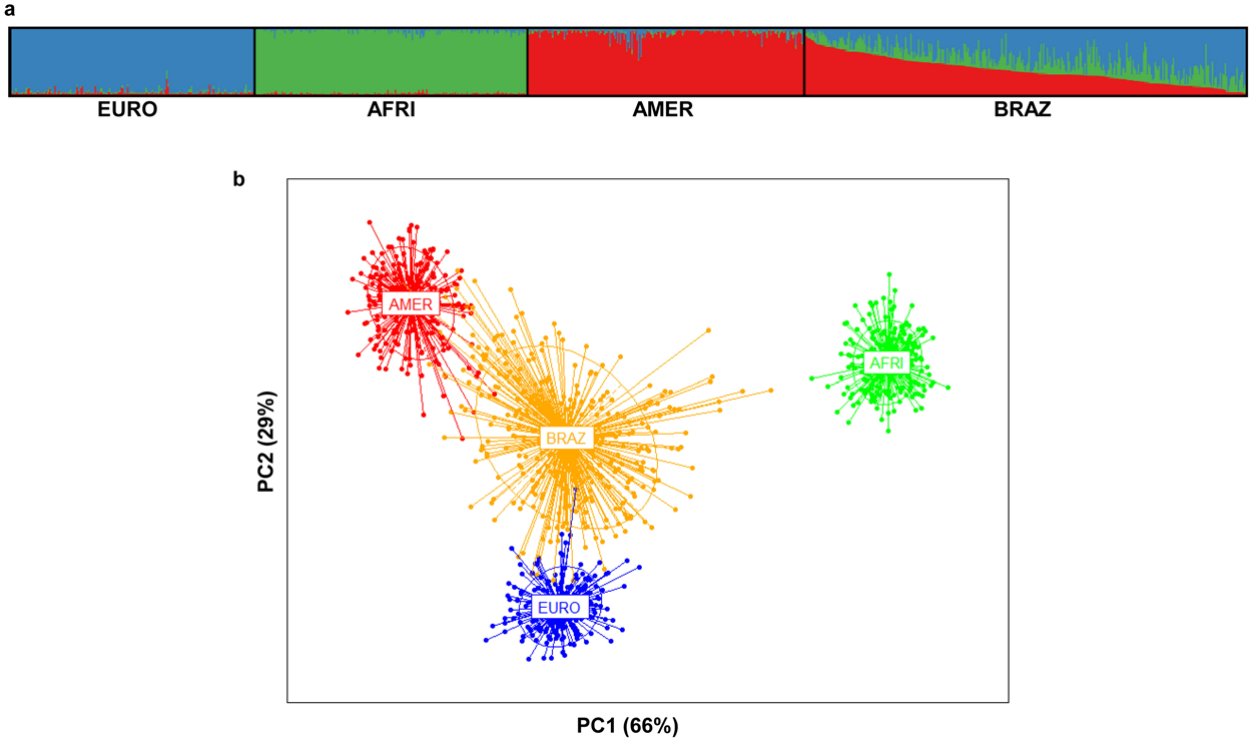
Genomic ancestry of the Rio Pardo population. (**a**) Genomic individual ancestry bar plot, including worldwide populations and the Rio Pardo population. Each individual is represented by a thin vertical line that is partitioned into three colored segments corresponding to the individual’s estimated membership proportion in the European (blue), African (green) and Native American (red) clusters. (**b**) PCA representation of individual ancestry. EURO, European; AMER, Native American; AFRI, African and BRAZ, Brazilian (Rio Pardo population).

The genomic ancestry of 339 unrelated subjects was estimated by genotyping a validated set of 48 AIMs dispersed in the human genome. The allele frequencies at these loci are shown in Supplementary Table S3. One locus showed departure from Hardy–Weinberg equilibrium (HWE, P < 0.01) in the Rio Pardo population (Table S2). Population-based analyses indicated that the Rio Pardo population has strong departure of Hardy–Weinberg equilibrium (*F*_IT_ = 0.04, 95% CI 0.02–0.06), which could be the result of the high level of inbreeding estimated for this population (*F*_IS_ = 0.03, *P* = 0.015) and ancestry-based population subdivision (ρ_FIT-FST_ = 0.33, *P* = 0.022). The Bayesian cluster approach to inferring the genetic population structure showed that the AIMs used discriminated among European, African and Native American parental populations (Fig. 1a). The mean estimates of ancestry for the individuals from the Rio Pardo settlement pointed to a highly admixed population with significant contribution of European (44.1%, 95% CI 41.8–46.5) and Native American (37.6%, 95% CI 35.4–39.8) ancestries, but with low African ancestry (18.3%, 95% CI 16.9–19.7). Individual ancestry inference was confirmed by PCA analysis, which placed the Rio Pardo population between Europeans and Native Americans (Fig. 1b). The first two principal components (PCs) account for 95% of the variance, where PC1 clearly separates the Africans from all other groups, and PC2 clearly separates the Europeans from Native Americans. Because this set of AIMs was originally selected to maximize the differences among European Americans, Africans and Native Americans, it was not surprising that both approaches clearly distinguished the three parental populations^30^.

### Distribution of DARC Genotypes Among Subjects from the Rio Pardo Settlement According to Genomic Ancestry

Initially, we determined the DARC genotypes of 690 subjects from the Rio Pardo population and found a high prevalence of FY*A/FY*B (29.6%), FY*A/FY*A (23.2%) and FY*A/FY*O (20.1%) genotypes (Table 1). On the other hand, the FY*O/FY*O genotype was observed at a very low frequency (3.0%). In general, there was a predominance of the functional DARC alleles FY*A (48.0%) and FY*B (33.6%) in the study population. This locus was not found to be in HWE in the Rio Pardo population (*P* = 0.035), with a deficiency in the heterozygous DARC genotypes (60.3%) compared with what was expected (62.3%). Similarly, analysis with a subset of unrelated subjects also showed that this locus was not in HWE (*H*_O_ = 57.0% and *H*_E_ = 63.0%, *P* = 0.0005).

**Table 1:** Demographic, epidemiological and genetic data of 690 subjects from the Rio Pardo Settlement, Amazonas, Brazil.

Variables	
**Median age, years (IQR** ^**a**^ **)**	25 (11–45)
**Number of malaria episodes in 7 years** ^**b**^ **, median (Range)**	0 (0–24)
**Years of residence in the Brazilian Amazon Region, median (IQR)**	22 (11–38)
**Riverine population, n (%)**	245 (35.5)
**DARC genotypes, n (%)**	
FY*A/FY*B	204 (29.56)
FY*A/FY*A	160 (23.19)
FY*A/FY*O	139 (20.14)
FY*B/FY*B	93 (13.48)
FY*B/FY*O	73 (10.58)
FY*O/FY*O	21 (3.04)
**DARC alleles (%)**	
FY*A	48.04
FY*B	33.55
FY*O	18.41

^a^IQR, interquartile range.

^b^The number of malaria episodes was obtained from the Epidemiological Surveillance System for Malaria (SIVEP-Malaria) for the period between 2003 and 2009.

**Figure 2:**
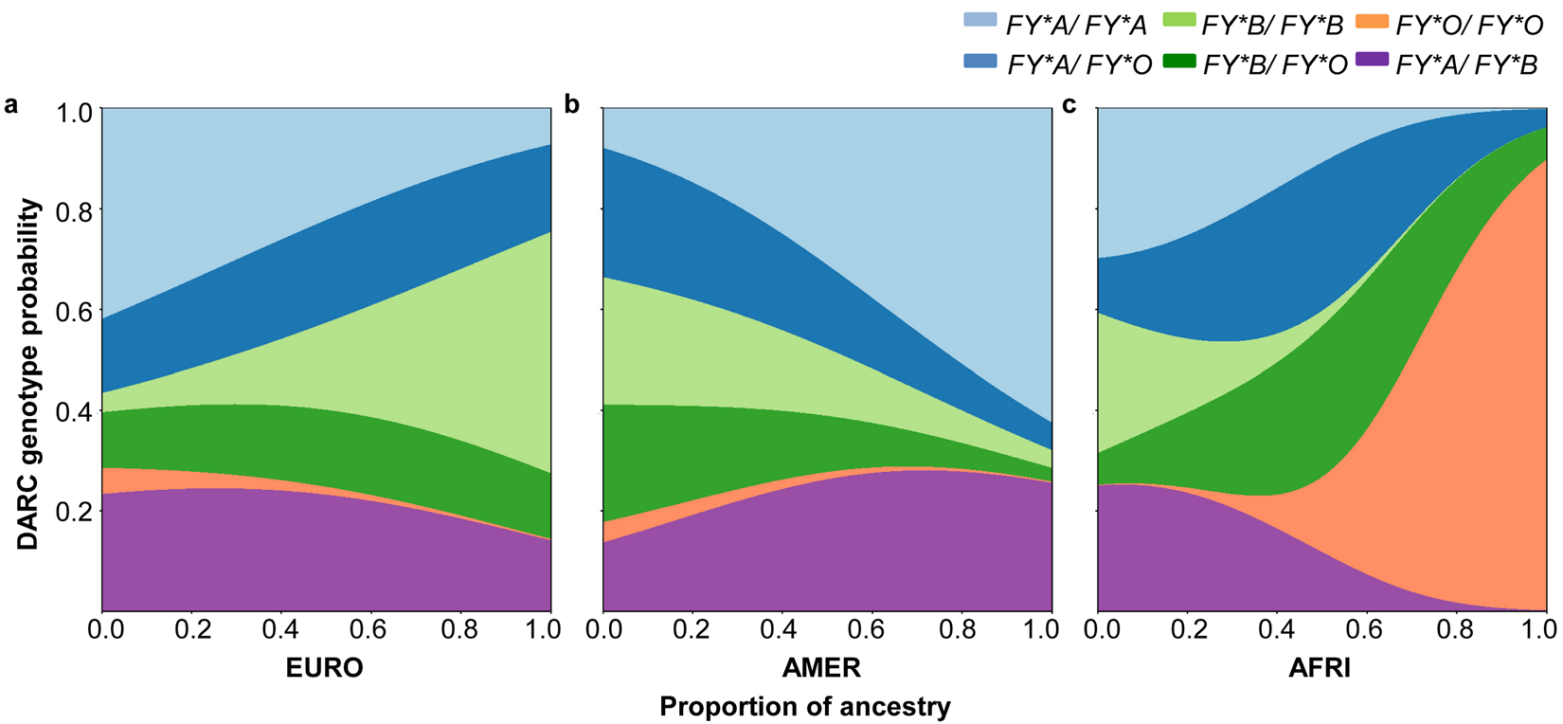
Frequency distribution of DARC genotypes according to the estimated genomic ancestry for the Rio Pardo population. Fitted multinomial logistic regression models showing the association between the distribution of DARC genotypes and (**a**) European, (**b**) Native American and (**c**) African ancestry. The individual proportion of genomic ancestry is shown in the x-axis. The y-axis is labeled on the probability scale and shows the variation in the probability of a genotype along with the variation in ancestry proportions.

We further described the association between DARC genotypes and the estimated individual genomic ancestry by fitting a multinomial logistic regression. The odds of having the FY*B/FY*B [relative risk ratio (RRR): 20.7; 95% CI 3.7–115.9; *P* = 0.001] and FY*A/FY*A (RRR 4.2; 95% CI 0.9–19.1; *P* = 0.06) genotypes increased continuously with the increase in the European and Native American ancestry, respectively, compared with the FY*A/FY*B genotype (Fig. 2a,b). An opposite trend was observed for FY*O allele carriers (RRR 0.11; 95% CI 0.02–0.64; *P* = 0.013 for FY*A/FY*O; RRR 0.06; 95% CI 0.01–0.43; *P* = 0.005 for FY*B/FY*O) and the FY*B/FY*B (RRR 0.08; 95% CI 0.01–0.47; *P* = 0.006) genotype to Native American ancestry. As expected, the probability of having the FY*O/FY*O genotype increased as the individual proportion of African ancestry increased (RRR > 1000; *P* = 0.00) (Fig. 2c).

### DARC Affects the Susceptibility to *Plasmodium vivax* malaria, as influenced by the Time of Malaria Exposure

To evaluate whether DARC has influenced the susceptibility to clinical *P. vivax* malaria the number of malaria episodes was recorded for each of the 690 participants during a period of seven years (2003–2009). The median number of episodes was zero but showed great variation (Range = 0–24) (Table 1). During the cross-sectional surveys (2008–2009), the overall prevalence of malaria (as detected by both microscopy and real-time PCR) was 7.0% (35 of 498) at baseline, with 89% of infections caused by *P. vivax*. At the 2nd and 3rd surveys, the overall frequencies of malaria infection were 1.6% (8 of 495) and 7.5% (37 of 495), respectively. All infections were caused by *P. vivax* at the 2nd survey, while 89% of infections were caused by *P. vivax* at the 3rd survey. No *P. malariae* or mixed *Plasmodium* infections were diagnosed by either microscopy or real-time PCR in the three cross-sectional surveys. The individuals had an average age of 25 years (IQR = 11–45) and lived for 22 years (IQR = 11–38) in the malaria endemic area (Brazilian Amazon region). In the Rio Pardo community, the time of residence was shorter (median = 6 years), partly reflecting the recent establishment of the settlement (approximately 20 years).

Next, we sought to assess the potential influence of demographic, epidemiologic and genetic factors on the *P. vivax* malaria incidence expressed as the total number of malaria episodes experienced per individual in a period of seven years. Three hundred twenty-two (47% of 690) subjects had at least one symptomatic *P. vivax* malaria episode between 2003 and 2009 (Supplementary Table S4). The number of malaria episodes was inversely and weakly correlated with age (ρ = −0.10, *P* = 0.010) and the time of residence in the Amazon region (ρ = −0.09, *P* = 0.018). As expected, because this was a stable population established mainly by migrants from the Amazon region, age was highly correlated with the time of residence in the Amazon region (ρ = 0.94, *P* < 0.001). Furthermore, a previous study in the community suggested spatial clustering of transmission, with a higher risk of malaria infection among people living along and around the local streams (riverine population)^26^.

**Figure 3:**
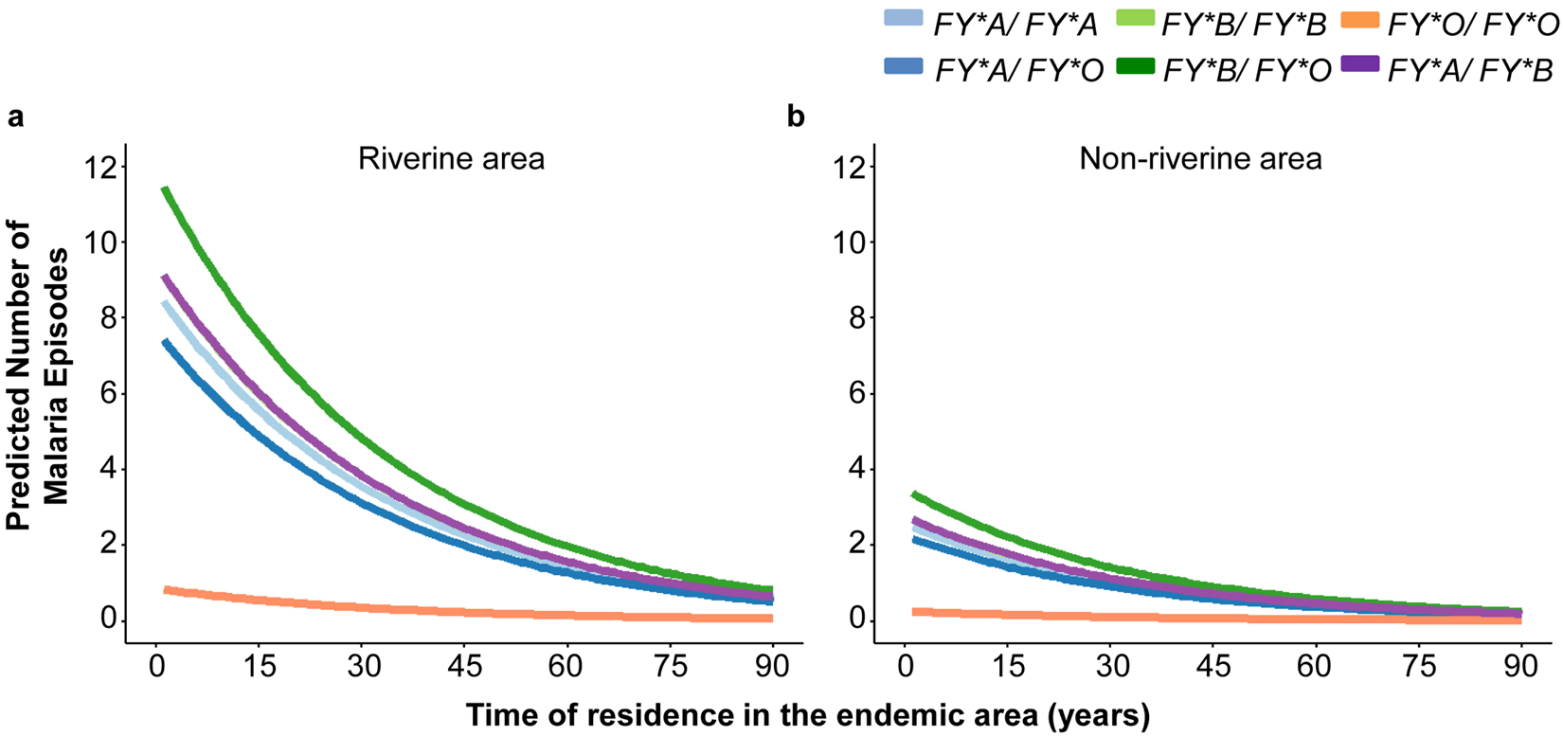
Analysis of the risk of clinical *P. vivax* malaria for the Rio Pardo population. The predicted number of clinical malaria episodes according to the DARC genotype, time of residence in the Amazon region and place of residence in the Rio Pardo community were derived from the Zero-inflated Poisson regression model. (**a**) Area along or close to the Rio Pardo stream with a higher risk of malaria infection (Riverine area) and (**b**) Non-riverine area with a lower risk of malaria infection.

Zero-inflated Poisson Regression (ZIP) analysis adjusted for the place of residence and time of residence in the endemic area showed a reduction in the risk of clinical *P. vivax* malaria of 19% (95% CI 2–32; *P* = 0.029) and 91% (95% CI 67–97; *P* = 0.0003), respectively, for subjects with the FY*A/FY*O and FY*O/FY*O genotypes compared with those with the FY*A/FY*B genotype (Table 2). By contrast, FY*B/FY*O individuals showed a greater risk (26%, 95% CI 3–53, *P* = 0.023) of clinical malaria compared with those with the reference genotype FY*A/FY*B. Of importance, the difference in susceptibility to malaria among DARC genotypes was decreased according to the increase in residence time in the endemic area (Fig. 3a,b). We observed a reduction of 3% (95% CI 2.5–3.4; *P* < 0.0001) in the risk for *P. vivax* malaria per additional year of residence in the endemic area and a variable risk depending on the transmission level in the place of residence (Fig. 3a,b; Supplementary Table S5). For instance, the riverine population was at an increased risk of *P. vivax* malaria (Fig. 3a).

**Table 2:** Effect of the DARC genotypes on the risk of clinical *P. vivax* malaria.

DARC Genotype	Relative Risk (95% CI)^a^	*P* ^b^
FY*A/FY*A	0.92 (0.78–1.10)	0.366
FY*A/FY*O	0.81 (0.68–0.98)	**0.029**
FY*O/FY*O	0.09 (0.03–0.33)	**0.0003**
FY*A/FY*B	*Reference*	
FY*B/ FY*B	1.00 (0.81–1.24)	0.993
FY*B/FY*O	1.26 (1.03–1.53)	**0.023**

^a^The relative risk of clinical malaria was estimated by Zero-inflated Poisson Regression adjusting for the place of residence and time of residence in the endemic area.

^b^The text in bold indicates that the relative risk is statistically significant.

### Antibody response varies with the DARC genotype

We next investigated whether the association between DARC variants and risk of *P. vivax* malaria was reflected in the acquisition of IgG total antibodies against blood-stage antigens. Here, to obtain a more comprehensive understanding of naturally acquired *P. vivax* immunity, we evaluated the antibody response against two leading blood-stage *P. vivax* vaccine candidates spanning a range of immunogenicity, the PvDBP_II_ and PvMSP1_19_ antigens. Antigen-specific total IgG levels were measured for each subject at baseline and after 6 and 12 months. The anti-PvDBP_II_ response was evaluated for 30% (207/690) of subjects who participated in the three cross-sectional surveys. The median antibody response measured by RI at baseline, 6 months and 12 months were 0.50 (IQR = 0.21–2.12), 0.47 (IQR = 0.22–1.78) and 0.57 (IQR = 0.24–1.96), respectively (Kruskal–Wallis test, *P* = 0,695). We found no association between the antibody response against PvDBP_II_ and DARC variants in this population (Supplementary Table S6).

**Figure 4:**
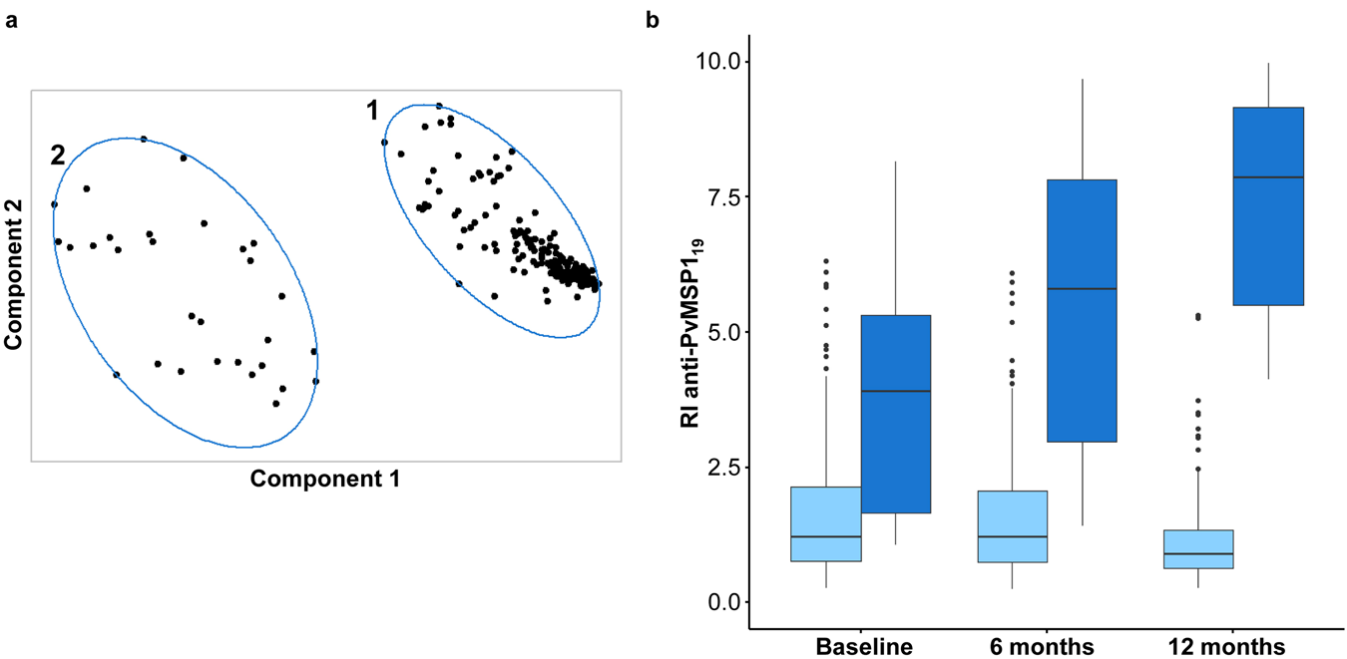
IgG total antibody levels against PvMSP1_19_ during the 12-month follow-up study in the Rio Pardo Settlement. (**a**) Individuals were clustered according to the anti-PvMSP1_19_ antibody levels evaluated in three cross-sectional surveys (baseline, 6 months and 12 months): Group 1: low level of response; Group 2: high level of response. The two components explained 90% of the point variability. (**b**) Anti-PvMSP1_19_ antibody levels in the follow up for the low-response (light blue) and high-response (dark blue) groups. The results were expressed as the reactivity index (RI).

Overall, the anti-PvMSP1_19_ response was evaluated for 32% (222/690) of participants in the three cross-sectional surveys. The median antibody responses measured by RI at baseline, 6 months and 12 months were 1.35 (IQR = 0.80–2.50), 1.37 (IQR = 0.79–2.30) and 1.03 (IQR = 0.67–2.03), respectively (Kruskal–Wallis test, *P* = 0.007). An association between the antibody response against PvMSP1_19_ and DARC variants was found at 6 months (Supplementary Table S7). We performed clustering analysis using the anti-PvMSP1_19_ antibody levels measured in the three cross-sectional surveys to allow more consistent identification of the different profiles of antibody responders considering that the levels of malaria transmission fluctuated in the study area. Individuals were clustered into two groups, the low (group 1, N = 166) and high (group 2, N = 30) antibody level group (Fig. 4a). The frequency of non-responders to PvMSP1_19_ (RI < 1.0) in the low-response group varied between 42% and 57% across surveys. Both the frequency and magnitude of the antibody response to PvDBP_II_ was lower than those of the anti-PvMSP1_19_ response in the three cross-sectional surveys (Table 3 and Fig. 4b). Due the differences in the immunogenicity of these merozoite proteins, clustering analysis was performed for both antigens separately. The high-response group included older individuals who have lived for a longer time in the malaria-endemic area and have experienced a higher number of malaria episodes than the low-response group (Table 3). In addition, 50% of subjects from the high-response group have experienced a *P. vivax* malaria episode recently (Table 3). We found a higher proportion of individuals from the high-response group living close to the river (Riverine area) (Table 3). The frequency and levels of anti-PvDBP_II_ antibodies were significantly different between the two groups, probably due to differences in malaria exposure between the groups (Table 3). We previously demonstrated that the subject’s age, which was correlated with cumulative exposure to malaria in the study population, was a strong predictor of seropositivity to PvDBP_II_^26^. Additionally, the distribution of the frequency of the DARC genotypes differed significantly between the low- and high-response groups (Table 3).

**Table 3:** Demographic data of the individuals clustered according to the anti-PvMSP1_19_ antibody response.

Variables	Group		*P*-value
	Low-Response (n = 166)	High-Response (n = 30)	
**Median Age, years (IQR)**	29 (13–48)	49 (23–56)	**0.004** ^a^
**Years of residence in the Brazilian Amazon Region, median (IQR)**	24 (12–43)	41 (20–55)	**0.003** ^a^
**Number of malaria episodes in 7 years, median (IQR)**	1 (0–3)	2 (1–4)	**0.037** ^a^
**Recent** ***P. vivax*** **infection, n (%)**			
Yes	43 (26)	15 (50)	**0.015** ^b^
**Local of residence in Rio Pardo, n (%)**			
Non-riverine	106 (64)	13 (43)	
Riverine	60 (36)	17 (57)	**0.042** ^b^
**DARC genotypes** ^c^ **, n (%)**			
FY*A/FY*B	54 (32.5)	3 (10.0)	
FY*A/FY*A	45 (27.1)	10 (33.3)	
FY*A/FY*O	36 (21.7)	9 (30.0)	
FY*B/FY*O	27 (16.3)	5 (16.7)	
FY*O/FY*O	4 (2.4)	3 (10.0)	**0.032** ^b^
**Antibody response** ^**d**^ **, (%)**			
**PvMSP1**_**19**_	43–58	100	
**PvDBP**_**II**_	29–33	60–76	
**RI PvDBP**_**II**_ **, median (IQR)**			
Baseline	0.42 (0.20–1.57)	2.57 (0.35–7.22)	**0.001** ^a^
6 months	0.41(0.20–1.32)	2.69 (0.40–6.67)	**<0.001** ^a^
12 months	0.45 (0.18–1.43)	4.61 (1.01–13.00)	**<0.001** ^a^

^a^Mann-Whitney U test.

^b^Fisher’s exact test.

^c^Individuals with the FY*B/FY*B genotype were excluded from these analyses due to restricted sample sizes.

^d^Percentage of individuals with a positive antibody response (RI > 1.0) across surveys. The sera reactivity was expressed as the reactivity index (RI) at 492 nm, with RI > 1.0 being considered positive.

**Figure 5:**
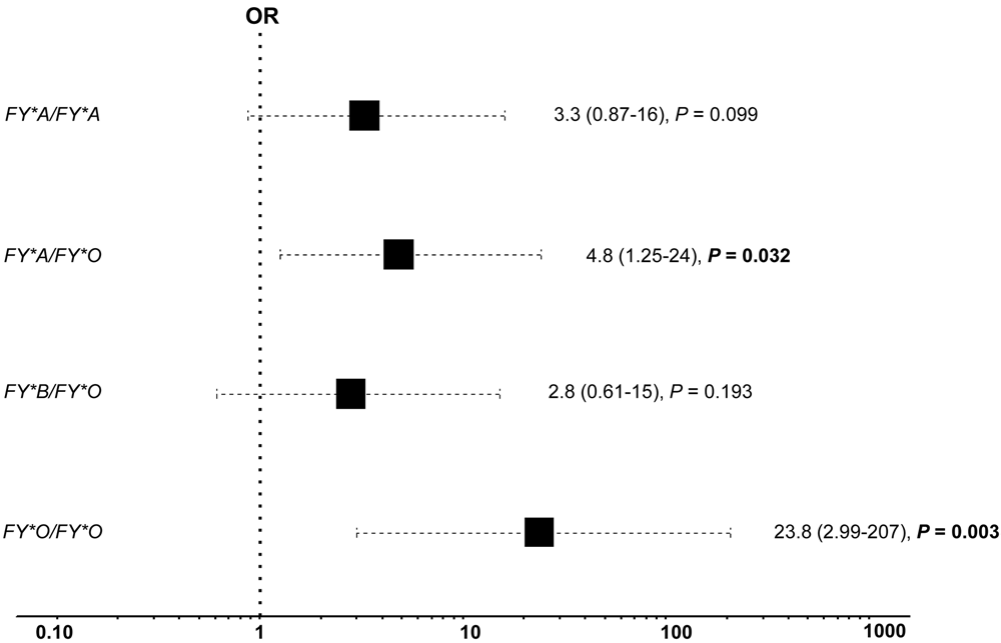
Association between the DARC genotype and antibody levels against PvMSP1_19_ during the 12-month follow-up study in the Rio Pardo Settlement. The logistic regression model was adjusted for the time of residence in the endemic area and recent *P. vivax* infection. Only variables associated with anti-PvMSP1_19_ antibody levels at a significance level of 0.05 (*P* < 0.05) were maintained in the final model. The odds ratio (OR, 95% CI) was adjusted using FY*A/FY*B as the reference group. The text in bold indicates that the OR is statistically significant.

**Figure 6:**
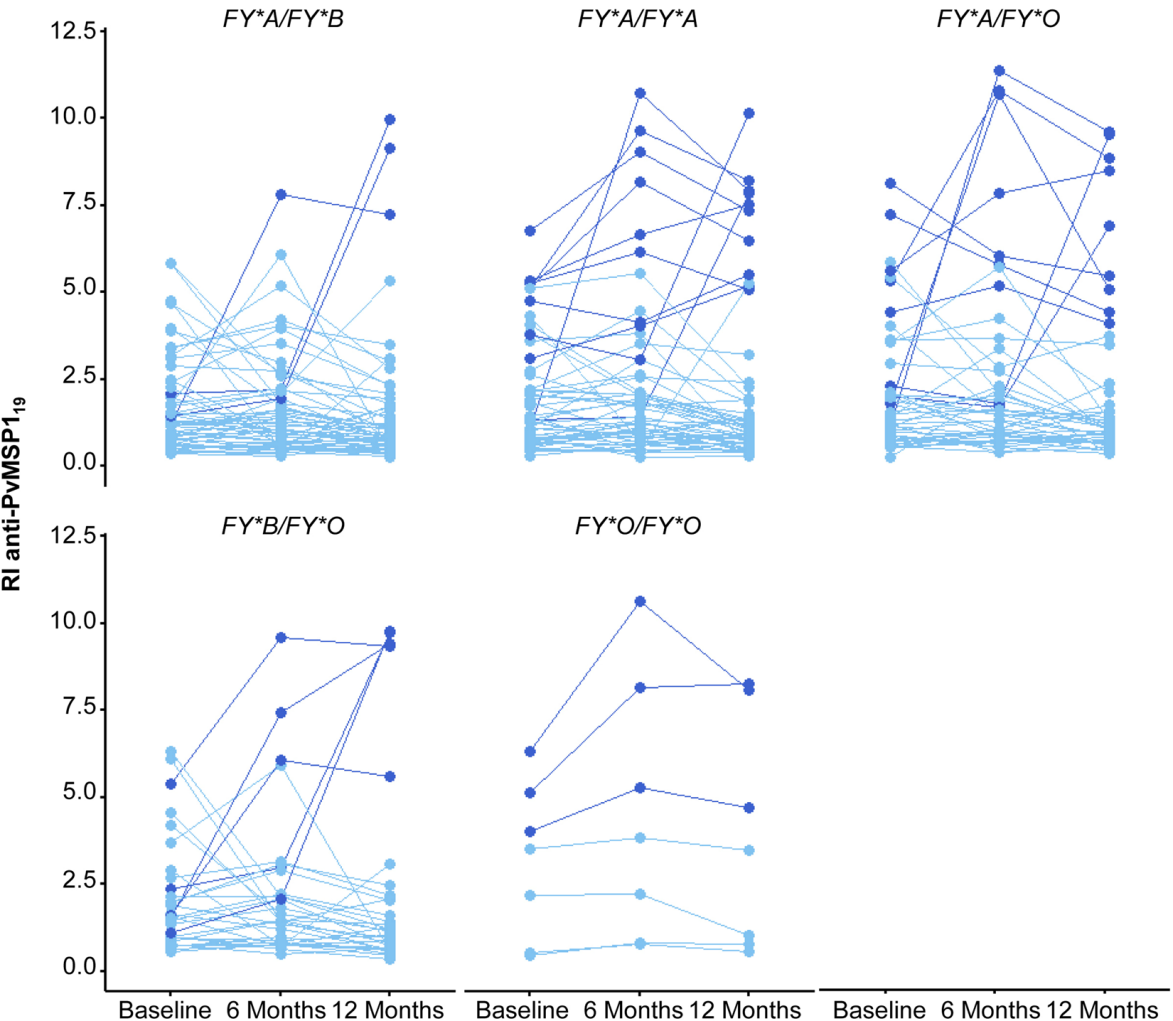
Individual anti-PvMSP1_19_ response evaluated in three cross-sectional surveys (baseline, 6 months and 12 months) according to the DARC genotype. The results were expressed as the reactivity index (RI). The antibody response data for low- and high-response groups are colored by light blue and dark blue, respectively.

The low- and high-response groups showed an anti-PvMSP1_19_ response significantly different in all three periods (Mann-Whitney U test, *P* < 0.001) (Fig. 4b). A logistic regression model was adjusted for the covariates: DARC genotype, number of malaria episodes, recent *P. vivax* malaria infection, time of residence in the Amazon region and place of residence in the Rio Pardo community. Nevertheless, the only variables significantly associated with anti-the PvMSP1_19_ antibody levels were the DARC genotype, recent *P. vivax* infection and time of residence in the endemic area. By fitting the logistic regression, individuals with the FY*A/FY*O genotype showed a higher chance (OR = 4.8; 95% CI 1.2–24; *P* = 0.032) to acquire high anti-PvMSP1_19_ antibody levels than the reference genotype FY*A/FY*B (Fig. 5). These individuals frequently showed high levels of the anti-PvMSP1_19_ antibody (Fig. 6). The FY*O/FY*O genotype was strongly associated with a high-response status (OR = 24, 95% CI 3–207; *P* = 0.003). This pattern of high antibody levels against PvMSP1_19_ was observed for all individuals with the FY*O/FY*O genotype, including individuals not included in logistic regression analysis due to missing data (Supplementary Fig. S1). Here, we observed that a longer period living in the malaria endemic area is associated with a slightly increased chance (OR = 1.04; 95% CI 1.02–1.07; *P* = 0.001) to acquire antibodies against PvMSP1_19_. In addition, individuals who had malaria recently (in the previous 6 months) showed an increased chance to develop anti-PvMSP1_19_ antibodies (OR = 4.8; 95% CI 1.9–13; *P* = 0.0008).

## Discussion

Malaria has provided a major selective pressure and has modulated the genetic diversity in the recent history of the human genome^52^. Growing evidence has pointed to erythrocyte variants that have resulted from evolutionary selection by malaria^53–55^. The variants of the *DARC* gene that apparently do not result in disease were probably selected by malaria parasites, particularly the FY*O allele^56^. Hence, most of the population of sub-Saharan Africa, where this allele is highly frequent, show the remarkable absence of *P. vivax* infection^6^. The ethnic differences in susceptibility and diverse genetic adaptations to *P. vivax* malaria for the other DARC variants are less clear. Here, we showed the influence of genomic ancestry on the distribution of DARC genotypes in the highly admixed Brazilian population and reinforced the recent finding that the FY*A/FY*O genotype is associated with decreased susceptibility to *P. vivax* malaria in a population-based follow-up. Conversely, the FY*B/FY*O individuals showed a greater risk of clinical *P. vivax* malaria. Of note, these differences in the susceptibility to clinical *P. vivax* malaria become more evident depending on the time of malaria exposure.

The distribution of DARC alleles shows a pattern of striking global divergence, whereas the FY*O allele is close to fixation (i.e., frequencies of 100%) in sub-Saharan Africa, and the most prevalent FY*A allele has become dominant across south-east Asia^57^. On the other hand, the ancestral FY*B allele is the least prevalent with a more limited distribution, being more frequent in Europe and parts of the Americas^57^. In the Americas, including Brazil, all three alleles are present, reflecting the great allelic heterogeneity. The highly heterogeneous Brazilian population was formed by the admixture between Native Amerindians, Europeans colonizers or immigrants, and African slaves^58^. Although the European component is preponderant in the Brazilian population (≈70%), with a slightly increasing trend from North to South of Brazil^59–61^, in the small settlement of Rio Pardo, a much lesser contribution of the European component (≈44%) was found. A significant Native American contribution (≈38%) was also estimated for this population, which comprises mostly natives of the Amazon region, mainly from the Amazonas State. Our data show that the high level of inbreeding and ancestry-based population subdivision are responsible to shape the genetic structure of this native population. Similar estimates of Native American ancestry were observed in individuals from the capital Manaus, which is located 160 km from the Rio Pardo settlement^62^. This genetic ancestry profile is consistent with the demographic history of northern Brazil, where land colonization and the genetic admixture process were different from other geographic regions of the country^63^. This process was characterized by the presence of numerous original indigenous populations^64^, social policies encouraging mating between European men and indigenous women during centuries of Brazilian colonization as a strategy for the occupation of the region, and the restricted use of African slave labor in the Amazon region^63,65,66^. Only after the 1960s occurred the frontier expansion in the Amazon as a result of the introduction of large-scale colonization projects and wide-ranging human settlement sponsored by the government^67^.

We further investigated the impact of ancestry on the distribution of DARC variants in the highly admixed population from the Rio Pardo community. A higher frequency of FY*A allele carriers and, consequently, the Fy^a+b−^ phenotype (i.e., FY*A/FY*A and FY*A/FY*O, 43%) was observed compared with the Fy^a-b+^ phenotype (i.e., FY*B/FY*B and FY*B/FY*O, 24%). A consistent finding is that the probability of having the FY*B/FY*B genotype or heterozygosity for the FY*O allele decreases with the increase in the Native American component. On the other hand, the probability of having the FY*B/FY*B genotype increases as the individual proportion of European ancestry increases. Considering that similar proportions of the European and Native American components together account for 82% of the diversity in individual genetic ancestry in the Rio Pardo population, a complex pattern of admixture with the predominant carriers of functional alleles FY*A and FY*B was found. Noticeably, this complexity is increased by the percentage of African ancestry present in individuals who are associated with a higher probability of carrying the FY*O allele. The frequency distribution of DARC genotypes described here differs from that reported in other populations in the Amazon region, except for the FY*A/FY*B and FY*O/FY*O genotypes, which are, respectively, the most frequent (≈30%) and rare genotypes (≈3%) in northern Brazil^15,68–70^. The main differences were found for the FY*A/FY*O and FY*B/FY*B genotypes (20% and 13% in the Rio Pardo population, respectively), which showed median frequencies of 12% (Range = 7–15) and 18% (Range = 14–22), respectively, in the Amazon region^15,68–70^. The differences in the genotype frequencies observed for the Amazon population reflects the global scenario of the ethnic heterogeneity of the Brazilian population. Interestingly, individuals from the Rio Pardo community did not fit Hardy–Weinberg expectation. Here, we considered two possibilities to explain this result: demographic disequilibrium due to inbreeding and natural selection acting on the DARC locus. Indeed, a high level of inbreeding was estimated for this population.

To account for a role of natural selection in the pattern of distribution of DARC variants, we investigated the possible association between DARC genotypes and *P. vivax* malaria epidemiology. In the study area, FY*A/FY*B was the most frequent genotype, followed by the FY*A/FY*A and FY*A/FY*O genotypes. For other areas of the Brazilian Amazon, FY*A/FY*B has also been described as the major variant^15,68,69^. Although the FY*O/FY*O frequency was very low in the study area, we found three of 21 individuals infected by *P. vivax* during the study period. Therefore, the FY*O/FY*O genotype was associated with a very low risk of clinical *P. vivax* malaria in the Rio Pardo community. *Plasmodium vivax* infection among DARC-negative individuals has been previously described in the Brazilian Amazon^71^. We also found a reduced risk of clinical malaria for individuals with the FY*A/FY*O genotype compared with those with the FY*A/FY*B. By contrast, FY*B/FY*O presented a greater risk to develop clinical *P. vivax* malaria. These results are in accordance with previous findings describing the effect of different DARC genotypes on the risk of clinical malaria in a northern Brazilian population^15^. In this previous study, individuals with the FY*A/FY*O genotype showed a higher magnitude of reduction regarding the risk of clinical malaria (80%) than those with the FY*A/FY*B genotype. Additionally, subjects with the FY*B/FY*B genotype showed a greater risk (270%) of clinical *P. vivax* malaria. The differences in the strength of association among DARC variants and clinical malaria observed between the studies may be related to epidemiological, demographic and genetic particularities of each population. In fact, we showed that the differences among DARC variants in the susceptibility to malaria become more evident according to the residence time in the endemic area and, therefore, may reflect the immune status to malaria of the subject. Accordingly, the population of the previous study was a frontier settlement constituted mainly by migrants from the malaria-free regions of Southeast and South Brazil^72^ who had been living for a median of 14 years in the Brazilian Amazon^19^. In this population, in contrast to what we observed in the Rio Pardo population, there was a higher proportion of the FY*B/FY*B and FY*B/FY*O genotypes in addition to the FY*A/FY*B genotype^15^. Considering the complexity of malaria epidemiology, we cannot rule out the possibility that other factors related to the parasite (e.g., *P. vivax* variants) or the host (e.g., immune response and genetic variants in the blood group systems) that were not considered in both studies could also be responsible for the observed differences. Furthermore, the analysis of a higher number of individuals may be necessary to evaluate the risk of clinical malaria among the DARC variants. Despite these limitations, the findings from both studies are consistent with a protective effect of the FY*A allele and increased susceptibility of FY*B.

An important point to be investigated was how susceptibility to *P. vivax* infection influenced by DARC has modulated the immune response to malaria antigens. We evaluated the antibody response against two leading *P. vivax* vaccine candidates, the PvDBP_II_ and PvMSP1_19_ antigens. Of importance, although both antigens are expressed during the blood-stage cycle, they show very different profiles of an antibody response that likely is influenced by the host and parasite features^26^. Here, we found no association between the conventional antibody response against PvDBP_II_, as detected by ELISA, and DARC variants. These results confirmed our previous findings in which the frequencies of ELISA-detected IgG antibodies were similar between individuals carrying one or two DARC functional alleles (FY*A and FY*B)^25^. However, the association between DARC variants and susceptibility to *P. vivax* malaria seems to be reflected in the acquisition of anti-PvMSP1_19_ antibodies. A major finding was that DARC-negative individuals had a greater chance to acquire high levels of anti-PvMSP1_19_ antibodies in the entire follow-up period than FY*A/FY*B individuals. This is an unexpected result because most *P. vivax* isolates do not proceed to the blood-stage cycle in DARC-negative individuals. Another interesting result was that FY*A/FY*O individuals had a higher chance to acquire high levels of anti-PvMSP1_19_ antibodies than FY*A/FY*B individuals, although FY*A/FY*O individuals showed a reduced risk to develop clinical malaria. Likewise, individuals with the FY*A/FY*O genotype from the Caribbean coast of Colombia showed a higher trend to acquire anti-PvMSP-1_19_ than those with the FY*A/FY*B genotype^24^. Until now, two studies have investigated the immune response to *P. vivax* antigens regarding the DARC variants. In the African-Colombian population from the Colombian Pacific coast, it was found that the N terminus of PvMSP1 (200 L fragment) was similarly recognized by DARC-positive and -negative individuals^73^. Conversely, the frequency and magnitude of anti-PvMSP1_19_ antibodies were higher in DARC-positive individuals from the Colombia’s Caribbean coast^24^. However, this result should be interpreted with caution because both the frequency and levels of the antibody response to PvMSP1_19_ were very low in the study population^24^.

The reasons for the association found between DARC genotypes (Fy*O/Fy*O and Fy*A/Fy*O) and the anti-PvMSP1_19_ antibody response are not clear; however, we have raised three not mutually exclusive possibilities. First, the host immune response may be activated during the liver stage of parasite development because MSP1 is expressed during this stage, as shown for *P. falciparum* and *P. berghei*^74,75^. Thus, although most of the *P. vivax* isolates do not infect DARC-negative reticulocytes, the host immune response might be activated and/or boosted during the liver stage. Another possibility is the occurrence of *P. vivax* parasites adapted to use other receptors for reticulocyte invasion in the study area. Recently, DNA expansion of PvDBP was described in DARC-negative *P. vivax* infections, suggesting that it may be selected to allow low-affinity binding to another receptor on DARC-negative erythrocytes^76^. Finally, the lowest binding between PvDBP and DARC has been shown for FY*O allele carriers, who express decreased amounts of DARC compared with carriers of two functional alleles^14,15,19,77^. Therefore, it is possible to speculate that the low efficiency of invasion of FY*A/FY*O reticulocytes by merozoites might increase PvMSP1_19_ exposure to the immune system and boost the anti-PvMSP1_19_ antibody response.

In conclusion, this community-based study provides concluding remarks about modulation of the susceptibility of *P. vivax* malaria in a hypo to mesoendemic area such as the Amazon Brazilian region. We have shown how genomic ancestry is related to the distribution frequency of DARC genotypes in a population formed mainly by natives from the Amazon and characterized by the high contribution of Native Amerindian ancestry. In this population with an increased frequency of the FY*A allele, our results are consistent with a significant protective effect of the FY*A/FY*O genotype. Conversely, the FY*B/FY*O genotype is associated with increased susceptibility to clinical *P. vivax* malaria. Of importance, the differences in the susceptibility to malaria among DARC variants seem to be influenced by the time of exposure to malaria. Additionally, our findings indicate that differences in the susceptibility to malaria among DARC variants may modulate the immune response to malaria blood antigens such as PvMSP1_19_. Together, these findings have important implications for vaccine development and indicate the need to consider the level of exposure to malaria to evaluate differences in DARC mediating *P. vivax* susceptibility.

## Electronic supplementary material

Supplementary Information

Supplementary Table 4

## Data Availability

The datasets generated and/or analyzed during the current study are available from the corresponding author on reasonable request.
