# Supplementary material for: Susceptibility to Plasmodium vivax malaria associated with DARC (Duffy antigen) polymorphisms is influenced by the time of exposure to malaria
Source: Sci Rep. 2018 Sep 14;8:13851. doi: 10.1038/s41598-018-32254-z (PMC6138695; doi:10.1038/s41598-018-32254-z)
Supplement: Supplementary file 1 — Supplementary Information [file 41598_2018_32254_MOESM1_ESM.pdf]

SUPPLEMENTARY INFORMATION

Susceptibility to *Plasmodium vivax* malaria associated with DARC (Duffy antigen) polymorphisms is influenced by the time of exposure to malaria

Flora Satiko Kano, Aracele Maria de Souza, Leticia de Menezes Torres, Marcelo Azevedo Costa, Flávia Alessandra Souza-Silva, Bruno Antônio Marinho Sanchez, Cor Jesus Fernandes Fontes, Irene Silva Soares, Cristiana Ferreira Alves de Brito, Luzia Helena Carvalho & Tais Nobrega Sousa

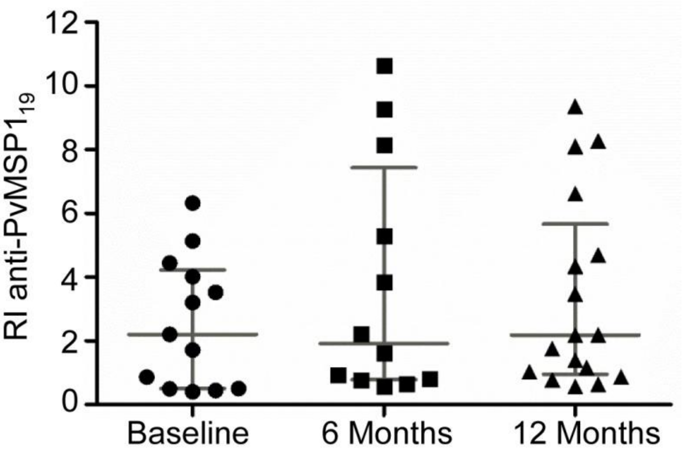

Supplementary Figure S1. IgG total antibody levels against PvMSP1$_{19}$ during the 12-month follow-up study for *FY*O/FY*O* individuals from the Rio Pardo Settlement. The anti-PvMSP1$_{19}$ response was evaluated at baseline, and after 6 and 12 months. The results were expressed as reactivity index (RI). Median and interquartile range at baseline (median = 2.2; IQR = 0.5-4.2), 6 months (median = 1.9; IQR = 0.8-7.4) and 12 months (median = 2.2; IQR = 0.9-5.7) are shown in the plot.

Supplementary Table S1. Description of the 48 ancestry informative markers genotyped for the Rio Pardo population.

rs Number	Applied Biosystems Assay	Alleles
rs4908343	C___2494120_10	A/G
rs3737576	C__27471358_10	C/T
rs7554936	C__26139689_10	C/T
rs260690	C____790944_10	A/C
rs6548616	C__29071253_10	C/T
rs9845457	C___1478361_10	A/G
rs10007810	C___1386349_10	A/G
rs7657799	C__29422763_10	G/T
rs870347	C___3052139_10	A/C
rs6451722	C___2938090_10	A/G
rs6422347	C__29040411_10	C/T
rs1040045	C___8767011_10	A/G
rs7803075	C___2130393_10	A/G
rs10108270	C__30263561_10	A/C
rs2416791	C__16234767_10	A/G
rs772262	C___8340116_10	A/G
rs9319336	C__27328815_10	C/T
rs7997709	C__30127919_10	C/T
rs9530435	C__27192660_10	C/T
rs9522149	C__30502208_20	C/T
rs3784230	C___2770233_10	A/G

rs11652805	C__31084340_10	C/T
rs4891825	C__27956007_10	A/G
rs4463276	C____493379_10	A/G
rs731257	C_____14517_10	A/G
rs4918842	C___3001048_10	C/T
rs2125345	C__15885530_10	C/T
rs4821004	C___2465604_1_	C/T
rs798443	C___8914321_10	A/G
rs10496971	C__30021395_20	G/T
rs9809104	C__30049893_10	C/T
rs1513181	C___1519732_10	A/G
rs316598	C___2949512_10	C/T
rs1325502	C___9030949_10	A/G
rs4666200	C__27891561_10	A/G
rs4670767	C__27978607_10	G/T
rs13400937	C__32187474_20	G/T
rs12629908	C___1508579_10	A/G
rs2504853	C__16252570_10	C/T
rs10236187	C____328256_10	A/C
rs3943253	C__11860358_10	A/G
rs1471939	C___8793707_20	C/T
rs4746136	C__27913671_10	A/G
rs2946788	C____302128_10	G/T
rs1760921	C___8833162_10	C/T
rs5768007	C__29548230_10	C/T

rs3907047	C___8948366_10	C/T
rs1837606	C___2910912_10	C/T

Supplementary Table S2. Description of oligonucleotides used for DARC genotyping by real-time PCR.

Oligonucleotide	Sequence
FGATANEW	5’-CCCGGGCCCGCCGCCCTCATTAGTCCTTGGCTCTTGC-3’
FAB	5’-CCCTCATTAGTCCTTGGCTCTTTT-3’
RABGATA	5’-AGGGGCATAGGGATAAGGGACT-3’
FY	5’-CTCAAGTCAGCTGGACTTCGAAGAT-3’
RYANEW	5’-AGCTGCTTCCAGGTTGGCGC-3’
RYB	5’-CTGCTTCCAGGTTGGCGT-3’

Supplementary Table S3. Allele frequencies of 48 loci genotyped for the Rio Pardo population.

	Frequency				HWE
**Locus**	**Allele 1**		**Allele 2**		***P*-value**
rs4908343	A	0.725	G	0.275	0.056
rs3737576	T	0.680	C	0.320	0.018
rs7554936	C	0.385	T	0.615	0.821
rs260690	A	0.534	C	0.466	0.656
rs6548616	C	0.372	T	0.628	0.082
rs9845457	A	0.596	G	0.404	0.501
rs10007810	A	0.382	G	0.618	0.487
rs7657799	G	0.213	T	0.787	0.105
rs870347	C	0.358	A	0.642	0.024
rs6451722	A	0.284	G	0.716	0.349
rs6422347	C	0.261	T	0.739	0.445
rs1040045	G	0.271	A	0.729	0.783
rs7803075	A	0.653	G	0.347	0.284
rs10108270	A	0.352	C	0.648	0.810
rs2416791	A	0.375	G	0.625	0.641
rs772262	A	0.393	G	0.607	0.494
rs9319336	C	0.345	T	0.655	0.070
rs7997709	C	0.381	T	0.619	0.160
rs9530435	C	0.740	T	0.260	0.674
rs9522149*****	C	0.376	T	0.624	0.000

rs3784230	G	0.513	A	0.487	0.326
rs11652805	C	0.364	T	0.636	0.410
rs4891825	A	0.688	G	0.312	0.800
rs4463276	A	0.625	G	0.375	0.200
rs731257	A	0.390	G	0.610	0.012
rs4918842	C	0.408	T	0.592	0.024
rs2125345	C	0.375	T	0.625	0.727
rs4821004	C	0.566	T	0.434	1.000
rs798443	A	0.561	G	0.439	0.015
rs10496971	G	0.328	T	0.672	0.109
rs9809104	C	0.371	T	0.629	0.010
rs1513181	A	0.395	G	0.605	0.365
rs316598	C	0.501	T	0.499	0.827
rs1325502	A	0.218	G	0.782	0.265
rs4666200	G	0.709	A	0.291	0.066
rs4670767	G	0.756	T	0.244	0.768
rs13400937	T	0.381	G	0.619	0.352
rs12629908	G	0.326	A	0.674	0.047
rs2504853	C	0.490	T	0.510	0.448
rs10236187	A	0.591	C	0.409	0.574
rs3943253	A	0.576	G	0.424	0.374
rs1471939	C	0.480	T	0.520	0.217
rs4746136	A	0.342	G	0.658	0.069
rs2946788	G	0.314	T	0.686	0.608
rs1760921	C	0.237	T	0.763	0.372

rs5768007	C	0.674	T	0.326	0.171
rs3907047	C	0.266	T	0.734	1.000
rs1837606	C	0.579	T	0.421	0.142

* The locus is not in Hardy-Weinberg equilibrium (HWE) in the Rio Pardo population ($P < 0.01$)

Supplementary Table S5. Effect of place and time of residence in the endemic area on the risk of clinical *P. vivax* malaria.

Variable	Relative Risk (95% CI)[a]	*P*[b]
Time of residence	0.971 (0.966-0.975)	< **0.0001**
Place of residence		
Non-riverine		
Principal	*Reference*	
Gusmão	0.960 (0.764-1.205)	0.723
Novo Paraíso	0.187 (0.088-0.398)	< **0.0001**
Terra Preta	0.201 (0.094-0.431)	< **0.0001**
Taxista	0.275 (0.111-0.683)	**0.005**
Riverine		
Samuel	1.306 (1.080-1.579)	**0.006**
Novo Progresso	1.743 (1.200-2.531)	**0.003**
Igarapé	2.433 (1.991-2.973)	< **0.0001**

[a] The relative risk of clinical malaria was estimated by Zero-inflated Poisson Regression adjusting for DARC genotype, place and time of residence in the endemic area.

[b] Text in bold indicates that relative risk is statistically significant.

Supplementary Table S6. Anti-PvDBP$_{II}$ antibody response according to DARC genotype for 207 participants in the three cross-sectional surveys.

DARC phenotypes	N[a]	RI PvDBP$_{II}$, median (IQR)		
		**Baseline**	**6 months**	**12 months**
*FY*A/ FY*B*	54	0.47 (0.19-1.46)	0.41 (0.21-1.26)	0.47 (0.20-1.94)
*FY*A/ FY*A*	50	0.57 (0.30-2.30)	0.69 (0.27-1.71)	0.71 (0.18-1.70)
*FY*A/ FY*O*	41	0.41 (0.23-1.14)	0.46 (0.23-1.80)	0.57 (0.30-3.75)
*FY*B/ FY*B*	23	0.71 (0.19-1.77)	0.59 (0.17-1.20)	0.61 (0.37-1.29)
*FY*B/ FY*O*	32	0.83 (0.24-2.90)	0.66 (0.22-2.44)	1.17 (0.30-4.24)
*FY*O/ FY*O*	7	0.28 (0.19-0.31)	0.32 (0.22-0.42)	0.19 (0.16-0.31)
P-value[b]		0.652	0.732	0.262

[a] Number of individuals

[b] Kruskal–Wallis test

Supplementary Table S7. Anti-PvMSP1$_{19}$ antibody response according to DARC genotype for 222 participants in the three cross-sectional surveys.

DARC phenotypes	N[a]	RI PvMSP1$_{19}$, median (IQR)		
		Baseline	6 months	12 months
*FY*A/ FY*B*	57	1.20 (0.77-2.11)	1.15 (0.70-2.09)	0.87 (0.60-1.69)
*FY*A/ FY*A*	55	1.35 (0.79-2.69)	1.41 (0.91-2.85)	1.03 (0.68-2.12)
*FY*A/ FY*O*	45	1.51 (0.86-2.95)	1.49 (0.90-2.86)	1.09 (0.76-2.37)
*FY*B/ FY*B*	26	1.23 (0.82-2.41)	1.05 (0.74-1.54)	0.85 (0.73-1.35)
*FY*B/ FY*O*	32	1.57 (0.95-2.44)	1.46 (0.81-2.39)	1.16 (0.66-2.09)
*FY*O/ FY*O*	7	3.53 (1.35-4.57)	3.85 (1.52-6.71)	3.47 (0.91-6.40)
P-value[b]		0.461	**0.043**	0.298

[a] Number of individuals

[b] Kruskal–Wallis test
